# Dung beetle-associated yeasts display multiple stress tolerance: a desirable trait of potential industrial strains

**DOI:** 10.1186/s12866-023-03044-z

**Published:** 2023-10-26

**Authors:** Anita Ejiro Nwaefuna, Mar Garcia-Aloy, Daniel Loeto, Thembekile Ncube, Andreas K. Gombert, Teun Boekhout, Saleh Alwasel, Nerve Zhou

**Affiliations:** 1 Department of Biological Sciences and Biotechnology, Botswana International University of Science and Technology, Private Bag 16, Palapye, Botswana; 2 Metabolomics Unit, Research and Innovation Centre, Fondazione Edmund Mach, Via E. Mach 1, 38098 San Michele All’Adige, Italy; 3 Department of Biological Sciences, University of Botswana, Private Bag, 0022 Gaborone, Botswana; 4 Department of Applied Biology and Biochemistry, National University of Science and Technology, P.O. Box AC 939, Ascot, Bulawayo, Zimbabwe; 5 School of Food Engineering, University of Campinas, Rua Monteiro Lobato 80, Campinas, SP 13083-862 Brazil; 6 Department of Zoology, College of Science, King Saud University, 11451 Riyadh, Saudi Arabia

**Keywords:** Stress-tolerant yeasts, Dung beetle-associated yeasts, Extremophiles, Non-conventional yeasts, Industry-associated stresses

## Abstract

**Background:**

Stress-tolerant yeasts are highly desirable for cost-effective bioprocessing. Several strategies have been documented to develop robust yeasts, such as genetic and metabolic engineering, artificial selection, and natural selection strategies, among others. However, the significant drawbacks of such techniques have motivated the exploration of naturally occurring stress-tolerant yeasts. We previously explored the biodiversity of non-conventional dung beetle-associated yeasts from extremophilic and pristine environments in Botswana (Nwaefuna AE et.al., Yeast, 2023). Here, we assessed their tolerance to industrially relevant stressors individually, such as elevated concentrations of osmolytes, organic acids, ethanol, and oxidizing agents, as well as elevated temperatures.

**Results:**

Our findings suggest that these dung beetle-associated yeasts tolerate various stresses comparable to those of the robust bioethanol yeast strain, *Saccharomyces cerevisiae* (Ethanol Red™). Fifty-six percent of the yeast isolates were tolerant of temperatures up to 42 °C, 12.4% of them could tolerate ethanol concentrations up to 9% (v/v), 43.2% of them were tolerant to formic acid concentrations up to 20 mM, 22.7% were tolerant to acetic acid concentrations up to 45 mM, 34.0% of them could tolerate hydrogen peroxide up to 7 mM, and 44.3% of the yeasts could tolerate osmotic stress up to 1.5 M.

**Conclusion:**

The ability to tolerate multiple stresses is a desirable trait in the selection of novel production strains for diverse biotechnological applications, such as bioethanol production. Our study shows that the exploration of natural diversity in the search for stress-tolerant yeasts is an appealing approach for the development of robust yeasts.

**Supplementary Information:**

The online version contains supplementary material available at 10.1186/s12866-023-03044-z.

## Introduction

Yeast species and strains are widely used in a variety of industries, including brewing, baking, winemaking, biofuel production, and other biomanufacturing industries that involve fermentation. However, the exposure of yeasts to harsh, habitat-irrelevant industrial conditions, characterized by various forms of environmental and metabolic stresses, such as high temperatures and the presence of osmotic, oxidative, and inhibitory compounds, is a major concern for industrial fermentation [1]. These stresses significantly affect cellular macromolecules, leading to inhibition of growth, an inability to survive, and subsequently a reduction in fermentative activity. The reduction in fermentative capacity of yeasts results in lower productivity and renders the production processes inefficient and unsustainable [2]. Therefore, the use of industrial strains with robust stress tolerance abilities is considered a cost-effective strategy that enables economically viable bioprocessing. Robust stress-tolerant industrial strains described and used in modern industry only account for a few yeasts, and most of them cannot withstand multiple stresses individually [3]. Despite extensive research into strategies aimed at improving the stress tolerance of industrial yeast strains, only a subset of robust strains are currently used in the fermentation industry [4]. Thus, the search for robust strains from biodiverse natural sources is appealing but remains largely unexplored.

Desirable traits for industrial production strains include the ability to tolerate high concentrations of ethanol, high temperatures, high osmotic stress, elevated oxidative stress, and inhibitory compounds [5]. Ethanol stress is caused by high ethanol concentrations, which inhibit substrate transport, cause DNA damage, denature enzymes, and disrupt the ion balance within yeast cells [6–8]. Osmotic stress leads to a membrane potential imbalance, which affects the activity of membrane transporters [9]. Excessive salt levels can result in hyperionic stress and disrupt the cellular ionic equilibrium [10]. The uncontrolled accumulation of reactive oxygen species (ROS), such as hydrogen peroxide (H_2_O_2_), superoxide anion (O_2_^−^), and hydroxyl radical (^•^OH), can induce oxidative stress [11–13]. ROS can damage various cell components, including DNA, mitochondria, the cytoskeleton, and proteins, contributing to yeast cell ageing and ultimately leading to cell death [2, 11–15]. High temperatures inhibit yeast cell growth and viability, disrupt cell membrane integrity, and denature ribosomes [16, 17]. Stress-tolerant yeasts are required to improve the fermentative performance of various industrial production processes, which are often inhibited by exposure to environmental and metabolic stressors.

Various strategies have been developed to increase the stress tolerance and robustness of industrial yeasts. These include the isolation and use of naturally stress-tolerant yeasts, the development of strains with robust stress tolerance abilities, and the removal of stressors in industrial processes, among others. Specific examples to improve the tolerance of existing yeast strains range from physiological to genetic strategies, such as evolutionary engineering [18–22], mutagenesis [23, 24], protoplast fusion [25, 26], mass mating, and genome shuffling [26–29]. The removal of stressors from the media or during the fermentation process has been employed in the removal of lignin derivative inhibitors by polymeric membranes (e.g., furfural, weak acids, and phenolic compounds) [30] to reduce the effects of these stressors. Many strategies, however, have their own limitations. For example, sexual hybridization as a means of strain improvement may not always be feasible due to certain strains exhibiting low sporulation efficiency, and some strategies such as direct mating can be time-consuming [4]. Therefore, cost-effective approaches for developing robust yeast strains capable of withstanding multiple environmental stresses are needed. Exploring natural yeast diversity in extreme habitats is an appealing approach to finding naturally stress-tolerant yeasts. We hypothesized that dung beetles might harbour yeasts with robust stress tolerance abilities due to their ability to inhabit extreme environments. Dung beetles that thrive in a lignocellulosic and extremophilic environment present an opportunity for discovering stress-tolerant yeast species [31, 32]. We ascertained that the insect gut can be regarded as hostile to microorganisms due to factors such as low pH, the presence of lytic enzymes, and the existence of an innate and cellular immune system [33]. Recently, we explored the biodiversity of yeasts associated with dung beetles inhabiting unexplored, pristine, and extremophilic environments characterized by semi-arid to arid and hot desert-like conditions in Botswana [31]. The extremophilic habitat of dung beetles could influence one of the yeast's most likely extremophilic life history strategies, which is its ability to tolerate a variety of stressors.

In this study, we evaluated the stress tolerance ability of 97 dung beetle-associated yeasts described in our previous work [31]. These yeasts were exposed to various stresses individually, such as high salt concentrations, high ethanol concentrations, high temperatures, formic acid stress, acetic acid stress, and oxidative stress, to assess their multiple stress tolerance abilities. Our results suggest that the non-conventional dung beetle-associated yeast isolates were tolerant to multiple stressors, such as high temperatures up to 42 °C, ethanol concentrations of up to 9% (v/v), formic acid concentrations up to 20 mM, acetic acid concentrations up to 45 mM, hydrogen peroxide up to 7 mM, and osmotic stress up to 1.5 M, comparable to one of the most robust industrial fermentation strains, Ethanol Red™ (*Saccharomyces cerevisiae*).

## Materials and methods

### Strains and media

A total of 97 non-*Saccharomyces* yeast strains were used in this study, which were previously isolated from dung beetles, as reported in our previous work [31]. The collection and identification of dung beetles, as well as the techniques used for isolating and identifying yeasts, were reported before. In this study, the yeast isolates were revived from − 80 °C freezers housed in the Department of Biological Sciences and Biotechnology at the Botswana International University of Science and Technology by plating them on yeast extract-peptone-dextrose (YPD) agar (1% yeast extract, 2% glucose, 2% peptone, and 2% agar, pH 6.2). The yeasts were streaked onto YPD agar and incubated (Thermo Scientific, MaxQ 6000, Ohio, USA) for three to five days. Afterwards, single colonies were selected for stress tolerance testing. YPD agar was used for cultivation and stress tolerance tests. The *S. cerevisiae*, Ethanol Red™ Version 1 strain (*Fermentis, Lesaffre,* France), a bioethanol yeast, was used as a control yeast, hereafter referred to as ER.

### Stress tolerance tests

To investigate the tolerance of dung beetle-associated yeasts to various stressors, the isolates were grown on YPD agar supplemented with different stressors. The phenotypic differences of the yeast isolates were determined using a spot plate assay approach, as described in [22]. In brief, the isolated yeast cells were grown overnight in test tubes containing 5 mL of YPD broth in an incubating shaker (Thermo Scientific, MaxQ 6000, Ohio, USA) at 30 °C and 180 rpm. To harvest the cells, the fermentation broth was centrifuged (Thermo Fisher Scientific, SL 16R, Germany) at 2 000 × g for 2 min, and the cells were then washed with 5 mL of sterile distilled water. The optical density (OD) of the cultures was measured at a wavelength of 600 nm using a spectrophotometer (VWR UV-1600PC, Pennsylvania, USA). The cells were adjusted to an OD_600nm_ of 0.2 and further diluted to OD_600nm_ of 0.1 and 0.05 in a 96-well plate containing sterile phosphate buffered saline (PBS). The dilutions were then spotted onto solid media plates containing the different stressors under investigation using an 8 × 6 replicator stamp (R2383, Sigma Aldrich).

To test for tolerance to acetic acid, formic acid, and ethanol, YPD agar was supplemented with different concentrations of the respective stressors, including acetic acid (40 mM, 45 mM, and 50 mM), formic acid (10 mM, 15 mM, and 20 mM), and ethanol (5%, 7%, and 9% (v/v)). Osmotolerance tests were performed using YPD agar enriched with sodium chloride (NaCl) (1 M and 1.5 M). To assess oxidative stress tolerance, hydrogen peroxide (H_2_O_2_) (3 mM, 5 mM, and 7 mM) was added as a supplement to the YPD agar. Before adding the stressors, the YPD agar was sterilized (121 °C for 15 min) and cooled to 50 °C–65 °C. Then, different concentrations of the stressors were added to the media, which was then poured into plates, allowed to cool, and spotted with yeast. All plates were incubated at 30 °C for 48 h. For thermotolerance testing, yeasts were spotted on YPD agar plates and incubated at 40 °C, 42 °C, and 44 °C for 48 h.

After a two-day incubation period, yeast growth on all test plates was examined. The best representative plates were selected from a total of nine plates, scanned (Epson Perfection V600 Photo Scanner, Suwa, Nagano, Japan), and analyzed. Three separate trial experiments were conducted per stressor, with each trial involving spot testing on three plates. As a result, a total of nine plates with similar outcomes were obtained after the three trials. Based on the scanned images, the growth of the yeasts was evaluated and scored on a scale ranging from 0 to 3, where: 0 = no growth (no growth detected on the plate); 1 = poor growth (growth detected on dilution 0.2 or slight growth detected in all dilutions); 2 = good growth (growth detected in dilution 0.2 and 0.1 or moderate growth detected in all dilutions); and 3 = excellent growth (visible growth detected in all dilutions). This evaluation was based on the performance of the isolates under different stress conditions, and the resulting data was presented as a heatmap. Principal component analysis (PCA) was further used to analyze the generated data, providing a complementary global perspective. The heatmap was generated using the R package “pheatmap” with raw data and default parameters, whereas the PCA was performed using the “prcomp” function, a standard component of the R software installation, after centering raw data. All experiments were conducted in triplicate and repeated three times.

## Results and discussion

### Dung beetle-associated yeasts exhibit multiple stress tolerance abilities

**Fig. 1:**
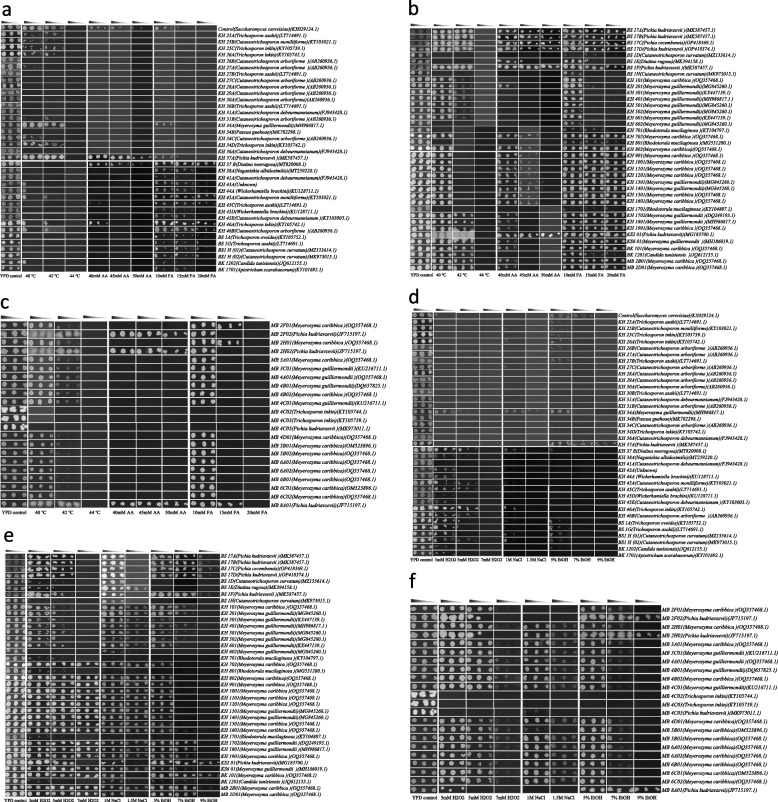
**a** Stress tolerance ability of dung beetle-associated yeasts. Ninety-seven dung beetle-associated yeasts were spot tested on YPD agar with varying formic acid (10 mM, 15 mM, and 20 mM) and acetic acid concentrations (40 mM, 45 mM, and 50 mM) to test for formic acid tolerance and acetic acid tolerance, respectively. The incubation temperature was set at 30 °C. The yeasts were also tested for thermotolerance on YPD agar and incubated at various high temperatures (40 °C, 42 °C, and 44 °C). The gradient shows the absorbance of the inoculum from left to right: 0.2, 0.1, and 0.05. The control *S. cerevisiae* (Ethanol red^TM^) was spotted on every plate. See also Additional file 1. **b** Stress tolerance ability of dung beetle-associated yeasts. Ninety-seven dung beetle-associated yeasts were spot tested on YPD agar with varying formic acid (10 mM, 15 mM, and 20 mM) and acetic acid concentrations (40 mM, 45 mM, and 50 mM) to test for formic acid tolerance and acetic acid tolerance, respectively. The incubation temperature was set at 30 °C. The yeasts were also tested for thermotolerance on YPD agar and incubated at various high temperatures (40 °C, 42 °C, and 44 °C). The gradient shows the absorbance of the inoculum from left to right: 0.2, 0.1, and 0.05. The control *S. cerevisiae *(Ethanol red^TM^) was spotted on every plate. See also Additional file 1. **c** Stress tolerance ability of dung beetle-associated yeasts. Ninety-seven dung beetle-associated yeasts were spot tested on YPD agar with varying formic acid (10 mM, 15 mM, and 20 mM) and acetic acid concentrations (40 mM, 45 mM, and 50 mM) to test for formic acid tolerance and acetic acid tolerance, respectively. The incubation temperature was set at 30 °C. The yeasts were also tested for thermotolerance on YPD agar and incubated at various high temperatures (40 °C, 42 °C, and 44 °C). The gradient shows the absorbance of the inoculum from left to right: 0.2, 0.1, and 0.05. The control *S. cerevisiae* (Ethanol red^TM^) was spotted on every plate. See also Additional file 1. **d** Stress tolerance ability of dung beetle-associated yeasts. Ninety-seven dung beetle-associated yeasts were spot tested on YPD agar with different hydrogen peroxide concentrations (H2O2) (3 mM, 5 mM, and 7 mM) for the oxidative stress tolerance test; different sodium chloride (NaCl) concentrations (1 M and 1.5 M) for the osmotolerance test and different ethanol concentrations (5%, 7%, and 9%) for the ethanol tolerance test. The incubation temperature was set at 30 °C. The gradient shows the absorbance of the inoculum from left to right: 0.2, 0.1, and 0.05. The control *S. cerevisiae* (Ethanol red^TM^) was spotted on every plate. See also Additional file 1. **e** Stress tolerance ability of dung beetle-associated yeasts. Ninety-seven dung beetle-associated yeasts were spot tested on YPD agar with different hydrogen peroxide concentrations (H2O2) (3 mM, 5 mM, and 7 mM) for the oxidative stress tolerance test; different sodium chloride (NaCl) concentrations (1 M and 1.5 M) for the osmotolerance test and different ethanol concentrations (5%, 7%, and 9%) for the ethanol tolerance test. The incubation temperature was set at 30 °C. The gradient shows the absorbance of the inoculum from left to right: 0.2, 0.1, and 0.05. The control *S. cerevisiae* (Ethanol red^TM^) was spotted on every plate. See also Additional file 1. **f** Stress tolerance ability of dung beetle-associated yeasts. Ninety-seven dung beetle-associated yeasts were spot tested on YPD agar with different hydrogen peroxide concentrations (H2O2) (3 mM, 5 mM, and 7 mM) for the oxidative stress tolerance test; different sodium chloride (NaCl) concentrations (1 M and 1.5 M) for the osmotolerance test and different ethanol concentrations (5%, 7%, and 9%) for the ethanol tolerance test. The incubation temperature was set at 30 °C. The gradient shows the absorbance of the inoculum from left to right: 0.2, 0.1, and 0.05. The control *S. cerevisiae* (Ethanol red^TM^) was spotted on every plate. See also Additional file 1

Ninety-seven yeast strains from various dung beetle species described by [31] were assessed for their ability to tolerate different stressors. Our findings revealed that most of the yeast isolates exhibited the ability to withstand various levels of stressors (Fig. 1a-f).

#### Thermotolerance

Thermotolerant yeast strains are essential for lowering cooling costs, reducing the risk of contamination, and safeguarding fermentation processes against failures caused by accidental thermal management errors or higher ambient temperatures [34, 35]. Furthermore, in the context of second-generation ethanol production, following the pre-treatment of lignocellulosic biomass, enzymatic hydrolysis is carried out at temperatures typically around 60 °C [36, 37], which is optimal for the enzymes used. The cooling time required for the process to be adequate for yeast inoculation is quite long. Therefore, the use of yeast strains capable of growth and fermentation at elevated temperatures could potentially increase productivity. Our findings suggest that some dung beetle-associated yeasts possess the ability to grow at high temperatures comparable to *S. cerevisiae* (ER). The results showed that 68.0% (66/97) of the yeast isolates grew at 40 °C, whereas 55.7% (54/97) of the yeasts, as well as the control yeast, grew at a maximum temperature of 42 °C (Fig. 1a-c). Perhaps the ability of these yeasts to grow at high temperatures may not be surprising, considering that the dung beetles were collected from Botswana, a country with environments where temperatures can reach up to 46 °C during the summer [38]. Most of the dung beetles were collected from northern Botswana, which is considerably warmer than the southern region. Therefore, these yeasts likely evolved special adaptations to survive under such conditions. Notably, *P. kudriavzevii (*KS2 01, MB 2F02, MB 2H02, MB 8A01, BS 17A, BS 17B, BS 17D, and BS 1F), *Pichia cecembensis* (BS 17C), *Meyerozyma guilliermondii* (KH 1702, KH 1801, KS6 01), and *Meyerozyma caribbica* (MB 2B01, BK101, KH 1901) grew exceptionally well at 42 °C (Fig. 1a-c). These findings are in agreement with previous studies that report thermal tolerance of the species *M. guilliermondii, M. caribbica,* and *P. kudriavzevii* at 40 °C [39, 40], and *P. kudriavzevii* at 41 °C [3] and 42 °C [41]. However, none of the isolated dung beetle-associated yeasts, including the control strain, were able to withstand a temperature as high as 44 °C. Nonetheless, our results demonstrate that dung beetle-associated yeasts possess thermotolerant abilities, which are important for high-temperature fermentation applications in the industry.

#### Tolerance to organic acid inhibitors

Industrial yeast strains that exhibit resistance to inhibitory or toxic compounds, whether they naturally occur in the medium or are produced as a by-product of fermentation, are highly desirable. In first-generation bioethanol production, the presence of contaminating bacteria, mainly lactobacilli, leads to acetic and lactic acid accumulation, both of which are detrimental to yeast [42–44]. Thus, it would be interesting to utilize yeasts that are tolerant to these compounds. Organic acids, such as formic acid and acetic acid, are known to inhibit cell growth and reduce the productivity of industrial fermentations, such as those in the brewing, winery, and biofuel industries [45, 46]. We evaluated the tolerance of dung beetle-associated yeasts to these inhibitors by assessing their growth at varying concentrations of organic acids.

Our results show that more than half of the yeasts tolerated formic acid concentrations of 10 mM (FA) (78.4% (76/97)) and 15 mM (FA) (54.6% (53/97)). The control yeast, *S. cerevisiae* (ER) only managed to grow in the former, thus suggesting that the dung beetle-associated yeasts were more tolerant to inhibitory formic acids (Fig. 1a). In addition, 43.2% (42/97) of the yeasts grew at the higher concentration of 20 mM (FA) (Fig. 1a-c). Notably, yeasts of the species *M. guilliermondii* and *M. caribbica* were able to grow well in formic acid concentrations as high as 20 mM, and their observed bigger colonies suggests that they may be able to tolerate even higher concentrations (not tested in this study) (Fig. 1a-c). On the other hand, up to 33.0% (32/97) of the yeasts were able to grow in concentrations of 40 mM acetic acid (AA), and 22.7% (22/97) could grow in concentrations as high as 45 mM (AA). Only 13.4% (13/97) of the total yeasts could tolerate acetic acid concentrations up to 50 mM. The control yeast, *S. cerevisiae* (ER), tolerated all the concentrations tested, albeit marginally, as demonstrated by the lower biomass, indicating its poor tolerance to inhibitory formic acid concentrations (Fig. 1a). *Pichia kudriavzevii* was one of the yeast species found to tolerate the highest concentrations of the stressors (Fig. 1a-c). *P. kudriavzevii* has been shown to tolerate high concentrations of acetic acid: 70 mM [47], 5 g/l (~ 83.3 mM) [41], and 100 mM [48], as well as formic acid at 30 mM [49]. Our findings suggest that dung beetle-associated yeasts are capable of withstanding exposure to inhibitory organic acids, which is crucial for their application in industrial fermentations.

#### Ethanol tolerance

Tolerance to high ethanol concentrations is essential for yeast cells in both their natural environment and in the industry [50]. As part of their typical metabolic processes, yeasts produce ethanol, which can be a stress-inducing agent [50] and may lead to inhibited cell growth, decreased cell viability, and reduced fermentation productivity [1, 8, 51]. Therefore, yeasts that are tolerant to elevated ethanol stress are attractive for cost-effective industrial fermentation processes. To test for tolerance to ethanol, different ethanol concentrations (i.e., 5%, 7%, and 9% (v/v)) were used. Our findings indicate that 74.2% (72/97) of the dung beetle yeasts were able to tolerate 5% ethanol (Fig. 1d-f). The control yeast, *S. cerevisiae* (ER) tolerated 5% and 7% (v/v) ethanol (Fig. 1d). However, dung beetle-associated yeast tolerance to ethanol decreased when exposed to higher concentrations of 7% and 9% (v/v). Specifically, 42.3% (41/97) of the total yeast isolates could tolerate 7% (v/v) ethanol, while only 12.4% (12/97) of the total yeasts tolerated a high ethanol concentration of 9% (v/v) (Fig. 1d-f). Among the yeasts that tolerated 9% (v/v) ethanol were *P. kudriavzevii* (KH 37 A, KS2 01, MB 2F02, MB 2H02, MB 8A01, BS 17A, BS 17B, and BS 1F), *P. cecembensis* (BS 17C), and *Meyerozyma caribbica* (BK 101 and MB 2B01) (Fig. 1d-f.). The ability of *P. kudriavzevii* to withstand elevated ethanol stress is well documented in the literature. The species has been shown to withstand ethanol levels of 12% [41], 13% [3, 52], 14% [53], and 15% [49, 54]. Our findings show the potential of dung beetle-associated yeasts to be used as potential yeasts where ethanol tolerance is desirable, e.g., for bioethanol production.

#### Oxidative stress tolerance

Oxidative stress is considered the most detrimental and fermentation-inhibiting stress encountered by yeast production strains during fermentation, leading to reduced fermentation efficiency [55]. Often, oxidative stress arises from an imbalance of oxygen-derived free radicals generated during fermentation [55], which can cause damage to cell components such as DNA and proteins [56]. It is noteworthy that high-temperature fermentation also increases oxidative stress [57]. Oxidative stress tolerance is therefore an important and desirable trait for strains used in industrial bioprocesses. Dung beetle-associated yeasts were grown at various H_2_O_2_ concentrations to test their tolerance to oxidative stress. Our findings indicate that 75.3% (73/97) of the dung beetle-associated yeasts tolerated a minimum H_2_O_2_ concentration of 3 mM, 67.0% (65/97) tolerated a H_2_O_2_ concentration of 5 mM, and 34.0% (33/97) tolerated a maximum H_2_O_2_ concentration of 7 mM (Fig. 1d-f). The control yeast, *S. cerevisiae* (ER), did not tolerate lower concentrations of H_2_O_2_ (Fig. 1d). Some yeasts exhibited a decrease in their tolerance abilities as H_2_O_2_ concentration increased, e.g., *Trichosporon ovoides* (BS1 A), *Trichosporon inkin* (KH 46 A), and *Diutina rugosa* (BS 1E), whereas others, such as *Cutaneotrichosporon arboriforme* (KH 25B), *Trichosporon asahii* (KH 30B), *Cutaneotrichosporon debeurmannianum* (KH 31A), and *Cutaneotrichosporon curvatum* (BS1 H), were unable to withstand oxidative stress caused by H_2_O_2_. Elevated H_2_O_2_ concentrations are toxic to yeast cells. A few yeast strains of *S. cerevisiae* [12, 58] and *P. kudriavzevii* have been shown to possess oxidative tolerance abilities [49, 59], whereas our findings suggest that dung beetle-associated yeasts have higher oxidative stress tolerance abilities.

#### Osmotolerance

Tolerance to osmotic stress is another essential trait of industrial strains, especially when the production medium contains high concentrations of sugar and/or salts. When yeast cells are exposed to environments with high sugar or salt concentrations, the osmotic pressure outside the yeast cells increases. This creates a gradient that draws water out of the cells, leading to cellular dehydration and subsequent osmotic stress [60–62]. Both sugar-induced and salt-induced osmotic stresses can have detrimental effects on yeast growth and metabolism. They can impair cell viability, hinder nutrient uptake, and affect the production of desired products or metabolites in industrial processes [60]. Therefore, osmotolerant yeasts would be extremely beneficial to the bioprocessing industry because they would improve yeast performance under osmotic stress [10]. Osmotolerant strains have mechanisms to maintain cellular integrity, water balance, and metabolic activity under high sugar and salt concentrations [63, 64]. To test their tolerance to osmotic stress, dung beetle-associated yeasts were grown at different NaCl concentrations (1 M and 1.5 M).

Our findings reveal that 72.2% (70/97) of the yeasts could withstand 1 M NaCl concentrations (Fig. 1d-f), whereas the control yeast, *S. cerevisiae* (ER), did not tolerate any of the NaCl concentrations (Fig. 1d). A similar study has revealed that an industrial *S. cerevisiae* strain, BG-1, is hypersensitive to sodium ions and cannot grow at 1 M NaCl concentration [58]. In the same study, other *S. cerevisiae* strains were able to grow at 1 M NaCl but could not tolerate NaCl concentrations exceeding 1.5 M. It is possible that the control yeast used in this study is also sensitive to sodium ions. The yeasts’ tolerance ability decreased to 44.3% (43/97) at a higher NaCl concentration of 1.5 M. *M. caribbica* and *M. guilliermondii* tolerated higher NaCl concentrations, as they grew efficiently in 1.5 M NaCl (Fig. 1d-f), because cells that adjust to slightly elevated osmolarity can withstand extreme osmotic shock [61]. Similarly, *M. caribbica* has been shown to tolerate NaCl concentrations of 1.5 M [65] and 2 M [3]. *M. guilliermondii* has also been shown to withstand up to 2.5 M NaCl [3]. As seen in our study, *P. kudriavzevii* yeast strains such as KS2 01, BS 1F, BS 17A, and *P. cecembensis* (BS 17C) exhibited osmotolerance abilities, as they were found to tolerate NaCl concentrations up to 1.5 M (Fig. 1d-f). Considering that the majority of yeasts were collected from northern Botswana [31], where saline environments are present [66, 67], it is likely that these yeasts have developed adaptations that aid their survival and allow them to withstand high salt concentrations. Our findings suggest that dung beetle-associated yeasts possess higher osmotolerance, an important trait sought after in bioprocessing industries.

Our results are in agreement with other studies that have shown that natural stress-tolerant yeasts can be isolated from various environmental sources, including fruit fermentations, nectar, soil, flowers, spontaneous wine fermentations, sugar cane juice, cheese, and contaminated beverages [3, 33, 54, 68–70]. These isolated stress-tolerant yeasts include thermotolerant yeasts such as *Kluyveromyces marxianus* [70–73] and *Pichia kudriavzevii* [41, 68, 74]*,* osmotolerant yeasts such as *Zygosaccharomyces rouxii* [60, 72, 75, 76] and *Torulaspora delbrueckii* [3, 77, 78], ethanol-tolerant yeasts such as *Dekkera bruxellensis* [79–81], and inhibitor-tolerant yeasts such as *P. kudriavzevii* [70, 81–83], *Zygosaccharomyces baili* [3, 70, 81], and *Wickerhamomyces anomalus* [52, 81].

### Dung beetle-associated yeasts with the ability to tolerate multiple stresses: a trait important for industrial applications

Various industrial processes, including traditional and commercial food fermentations such as baking, brewing, fermentations involving distilled products, and winemaking, expose cells to simultaneous or sequential combinations of different environmental stresses, making multi-stress resistance an attractive trait in industrial yeasts [84]. Therefore, multiple stress tolerance is a desirable trait that is essential for industrial production strains.

Yeast cells respond to environmental stresses by altering the expression of various genes, a process known as the environmental stress response (ESR) [85]. During fermentation, multiple signalling pathways and stress response genes regulated by different transcription factors coordinate a response to multiple stresses [86]. For example, heat shock genes regulated by heat shock transcription factor (HSF) 1 are induced not only by temperature shock but also by other stressful environmental changes such as ethanol stresses [87–89]. Our study reveals that dung beetle-associated yeasts have multiple stress tolerance abilities.

**Fig. 2:**
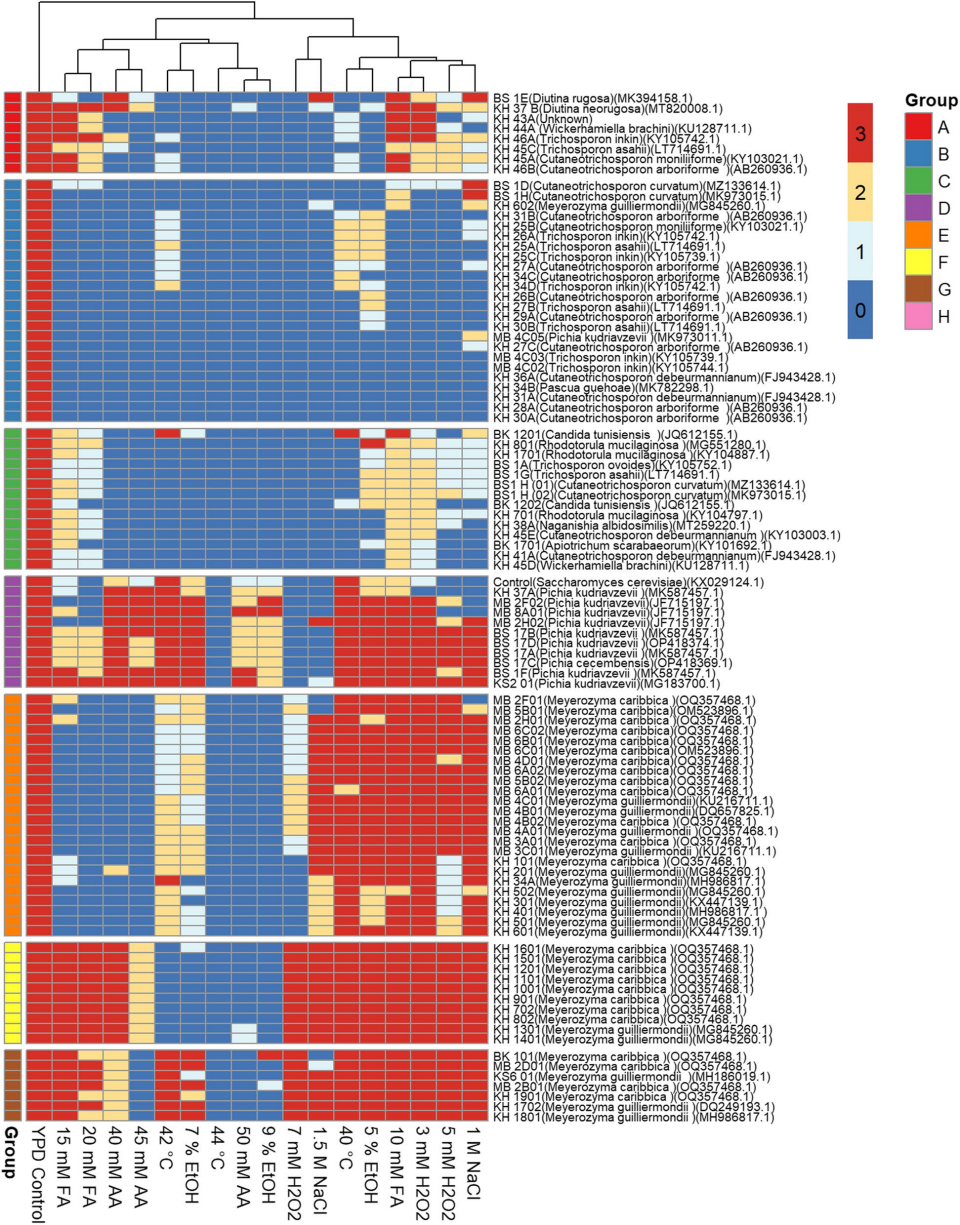
A heatmap depicting the scored growth performances of yeasts isolated from dung beetles under various stresses. The rows correspond to the 97 yeast isolates, while the columns correspond to the nine carbon sources used. The growth scores ranged from 0 to 3; 0 = no growth, 1 = poor, 2 = good, and 3 = excellent. The maximum possible score of 3 is represented in red, while the lowest possible score of 0 is given in blue. The yeasts are categorized based on how well they performed in each stress. AA = acetic acid, FA = formic acid, EtOH = ethanol, H2O2 = hydrogen peroxide, NaCl = sodium chloride. Yeasts grown in YPD agar were used as a control

A compilation of the results described above was organized in the form of a heatmap to enable the analysis of overall stress tolerance and to classify the isolated yeasts based on their ability to withstand different stressors (Fig. 2). The results reveal a huge disparity among isolates (Fig. 2, Additional file 1). Dung beetle-associated yeasts were categorized into seven specific groups (A-G) based on their tolerance to specific groups of stressors. The columns (i.e., stress conditions and YPD control) were clustered using Euclidean distance as a dissimilarity measure, whereas the rows (i.e., yeast isolates) were ordered using the output of Euclidean distance dissimilarity measures. However, the clusters generated were not indicated, as a slightly different grouping was chosen to facilitate the interpretation of the results.

Groups D, F, and G tolerated more stress than other yeasts (Fig. 2). These are the most appealing groups for the selection of industrial yeasts with multiple stress tolerances, despite their poor performance at some elevated stressor levels. When yeast cells are exposed to sublethal doses of one stress, they could become tolerant to high doses of that stressor and even against other different stresses they may not have been exposed to, also known as cross protection [8, 88]. Group G was distinguished by its ability to grow under all stress conditions except at an elevated temperature of 44 °C and acetic acid concentrations of 45 mM and 50 mM (Fig. 2). Except for growth at 42 °C, 44 °C, and 7% (v/v) and 9% (v/v) ethanol, Group F yeasts efficiently tolerated all stresses as well. Group D, consisting of isolates of *P. kudriavzevii* and *P. cecembensis*, performed well under all stress conditions except 44 °C and 7 mM H_2_O_2_. Several studies have reported that some *P. kudriavzevii* strains possess the ability to withstand multiple stresses [3, 41, 48, 49]. The control yeast in the same group grew moderately well. However, it could not tolerate 20 mM FA, 44 °C, 5 and 7 mM H_2_O_2,_ and 1 and 1.5 M NaCl. All yeasts in Group E could withstand temperatures as high as 42 °C, and most of them could tolerate H_2_O_2_ concentrations of up to 7 mM, NaCl concentrations of up to 1.5 M, and ethanol concentrations of up to 7% (Fig. 2). This group consisted of the *Meyerozyma* species.

Although most of the yeasts in this study exhibited multiple stress tolerance abilities, Groups A, B, and C showed patches of yeast with poor stress tolerance abilities. Most yeasts in Group A (mostly *Trichosporon* species) were able to withstand formic acid concentrations of up to 20 mM, ethanol concentrations of up to 5% (v/v), and NaCl concentrations of up to 1 M. However, less than half of the yeasts could withstand a temperature of 42 °C. Yeasts in Group B mostly grew efficiently in only YPD media, and some, including *T. inkin* (MB 4C02 and MB 4C03), *Pascua guehoae* (KH 34B)*, C. debeurmannianum* (KH 31A and KH 36A), and *C. arboriforme* (KH 28A and KH 30A), could not withstand any stress. As a result, they are unlikely to be considered for use in the fermentation industry. Yeasts from Group C grew at 20 mM FA, 1 M NaCl, and 5 mM H2O2, although poorly.

### Principal Component Analysis

**Fig. 3:**
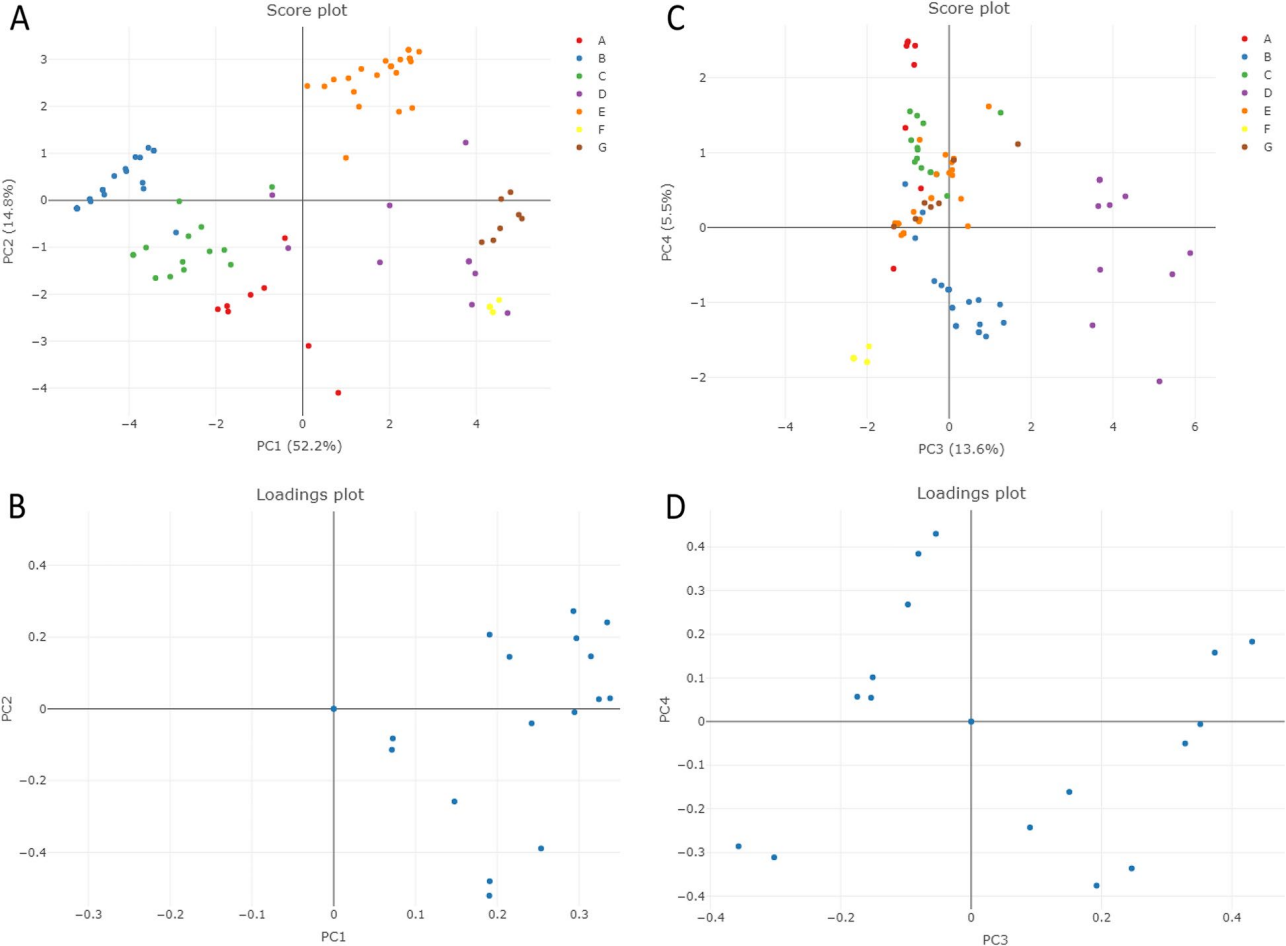
The score plot and corresponding loadings for principal component analysis (PCA) of the yeast groups from the heatmap data (Fig. 2). In each dimension, the value of the principal component is expressed as a percentage. **A** Score plot for PC1 & PC2, (**C**) Score plot for PC3 & PC4, (**B**) & (**D**) are loading plots of Group B from the heatmap, for (**A**) and (**C**) respectively

The output of the principal component analysis (PCA) (Fig. 3) supports what has been observed in the heatmap (Fig. 2). The defined groups based on the output of the heatmap are visible within the first two components of the PCA (Fig. 3). They effectively separated yeast groups A, B and C from groups E, F and G. Group B is notably separated from the others through the third component. On the score plot of the first two components of the PCA, all variables, except temperature at 44 °C (which resulted in the same value for all strains), are located on the same side of the graph, indicating that they are associated with groups E, F, and G. PCA components 3 and 4 further illustrate the separation of group B from the rest. This suggests that group B exhibits more resistance to stressors on the right side of the graph (e.g., 3, 5, and 7 mM H_2_O_2_; 40 °C; 1 and 1.5 M NaCl; 5 and 9% ethanol; and 10 mM formic (Fig. 2)) and less resistance to stressors on the left side (e.g., 15 and 20 mM formic acid; 40, 45, and 50 mM acetic acid; 7% ethanol; and 42 and 44 °C (Fig. 2)).

Taken altogether, our findings suggest that there are numerous dung beetle-associated yeasts from Botswana that can tolerate a variety of stresses. Although there are other strategies to develop multiple stress-tolerant yeasts for industrial applications, such as genetic engineering, evolutionary engineering, mutagenesis, and direct selection, they often suffer from many drawbacks (for reviews, see [21, 32]). In the food industry, the most important strategy remains the use of evolutionary engineering and the isolation of naturally robust strains from nature because consumers tend to prefer natural procedures over genetically manipulated technologies.

## Conclusion

Stress-tolerant yeasts are highly desirable for efficient and sustainable bioprocessing. Several strategies to develop robust yeast strains remain riddled with challenges. The isolation of yeasts from nature, possibly in combination with other techniques, could provide attractive avenues to obtain robust, stress-tolerant yeasts. This study has shed light on the potential of dung beetles as a source for multiple stress-tolerant yeasts. Interestingly, some of the dung beetle-associated yeasts exhibited superior performance compared to the control, *S. cerevisiae* Ethanol Red™ yeast, in various stress conditions, such as formic acid concentrations up to 20 mM, hydrogen peroxide up to 7 mM, and osmotic stress up to 1.5 M. Furthermore, it was observed that certain yeasts from the dung beetle samples exhibited exceptional growth at elevated temperatures of up to 42 °C, similar to the control yeast. In addition, some of these yeasts exhibited the ability to tolerate high ethanol concentrations of up to 9% and acetic acid concentrations of up to 50 mM, which were comparable to the control yeast. The search for robust yeasts from extremophilic environments in Botswana is the first of its kind. Future research in this area could focus on the in-depth characterization and optimization of these promising strains to fully exploit their potential for specific bioprocessing applications.

### Supplementary Information

**Additional file 1:** **Table S1. **Stress tolerance capability of dung-beetle associated yeasts examined using different stressors of varying concentrations. 

## Data Availability

The datasets supporting the conclusions of this article are included within the article and its additional files.
